# A Case of 22q11 Deletion Syndrome (22q11DS) with a Panayiotopoulos Epileptic Pattern: Are Additional Copy-Number Variations a Possible Second Hit in Modulating the 22q11DS Phenotype?

**DOI:** 10.3389/fped.2017.00048

**Published:** 2017-03-21

**Authors:** Veronica Bertini, Angelo Valetto, Alessia Azzarà, Annalisa Legitimo, Giuseppe Saggese, Rita Consolini, Alessandro Orsini, Alice Bonuccelli

**Affiliations:** ^1^Cytogenetics and Molecular Genetics Unit, Azienda Ospedaliero-Universitaria Pisana, Pisa, Italy; ^2^Laboratory of Immunology, University of Pisa, Pisa, Italy; ^3^Section of Pediatric Neurology, Azienda Ospedaliero-Universitaria Pisana, Pisa, Italy

**Keywords:** 22q11 deletion syndrome, epilepsy, array CGH, copy-number variations, 2q37, DGKD

## Abstract

“22q11 deletion syndrome” (22q11DS) is a rare genetic syndrome, in which most patients share the same deletion, but their clinical features may vary a great deal. The genetic mechanisms underlying the variable expressivity and reduced penetrance of 22q11DS still have to be fully elucidated. Epilepsy has been reported in about 15.2% of the patients; however, few studies have focused on this topic, and in most cases, a detailed epileptic profile is missing. Since only a minority of patients experience epileptic seizures, 22q11deletion can be considered a predisposing factor, which is not sufficient “*per se*” to cause epilepsy; to date, no candidate gene for epilepsy has been identified in the deleted region. We report on a 6-year-old girl with 22q11DS presenting a form of epilepsy that can be classified as “Panayiotopoulos syndrome.” Array CGH revealed an additional microduplication of 172 kb in 2q37, harboring three genes. One of these, *DGKD* (diacylglycerol kinase delta), is interrupted by the distal breakpoint of the duplication. *DGKD* encodes a cytoplasmic enzyme that phosphorylates diacylglycerol to produce phosphatidic acid. This is an important second messenger in a pathway of lipid signaling that has been implicated in epilepsy and other neurological diseases. Disruption of *DGKD* by a t(X;2) has been previously reported in a patient with epilepsy. The 2q37 microduplication was inherited from her mother, who never experienced epileptic seizures, thus this imbalance is not “*per se*” sufficient to cause epilepsy. It can be hypothesized that the epileptic phenotype is provoked by the simultaneous presence of 22q11.2 deletion and 2q37 duplication. It has been shown that rare additional copy-number variations (CNVs) outside the 22q11.2 region may modulate the risk of congenital heart defects. It is possible that also for the epileptic phenotype, the additional CNVs may represent an important modifying factor underlying the variable expressivity and incomplete penetrance in the 22q11DS.

## Background

“22q11 deletion syndrome” (22q11DS) is a rare genetic syndrome with a reported prevalence of about 1:4,000 live births. Although most of the patients share exactly the same deletion of nearly 3 Mb in 22q11.2, their clinical features may be highly variable ranging from a severe phenotype to a very mild one (1). The genetic mechanisms underlying the variable expressivity and reduced penetrance of 22q11DS still have to be fully elucidated.

Typical symptoms include multiple developmental anomalies such as congenital heart defects (CHD), palatal, renal, gastrointestinal, ophthalmological, dental, and skeletal abnormalities as well as hypocalcemia, immunodeficiency, and endocrine dysfunction (1). Structural central nervous system (CNS) abnormalities, like polymicrogyria, cerebral atrophy, cerebellar hypoplasia, focal cortical dysplasia, and subependymal heterotopia, are also reported (2). Neuropsychiatric (NP) manifestations such as neurodevelopment disorders (developmental delay, cognitive impairment, and speech disturbances) and psychiatric disorders (attention deficit hyperactivity disorder, schizophrenia, and mood disorders) are often present (2–4).

Among the NP manifestations, seizures are conspicuous events, but in the majority of cases, they can be a secondary manifestation of other syndrome-associated features, such as hypocalcemia, hypoxia/ischemia secondary to CHD, and fever with recurrent infections. In the literature, the epileptic seizure incidence is estimated from about 10% (5) to 21% (6). In a recent article, the incidence of epilepsy in a cohort of 145 22q11DS children was reported to be 15.2%; excluding patients with “structural epilepsy” (CNS abnormalities), the prevalence of “genetic epilepsy” is 8.3% (2).

These findings suggest a link between “genetic epilepsy” and 22q11DS, and this implies that the hemizygous deletion of one or more genes in this specific locus may be associated with epileptogenesis. However, 22q11.2 hemizygous deletion is not sufficient “*per se*” to cause epilepsy but constitutes a predisposing genetic factor, since only a minority of patients experience epileptic seizures. It may be that a “second hit” is necessary to manifest the epileptic phenotype, but to date, the genetic mechanisms that could increase the risk of epilepsy are not known.

Recently, array CGH has highlighted that humans are much more genetically variable than previously appreciated. To date, nearly 10–15% of the human genome has been annotated as harboring copy-number variations (CNVs), namely microdeletions and microduplications, ranging from 1 kb to several megabases (7). Their presence may contribute significantly toward human phenotypic variability, complex behavioral traits, and disease susceptibility.

Little is known about the contribution of additional CNVs on the phenotypic variability of 22q11DS. A recent article (8) focused on the role of rare CNVs as genetic modifiers predisposing to CHD in a very large cohort of patients with 22q11DS. When these CNVs were carefully examined for their gene content, specific cardiac networks appeared to be overrepresented in 22q11DS CHD cases but not in 22q11DS controls with no cardiac phenotype, suggesting that CNVs outside the 22q11.2 region can modify the risk for CHD in 22q11DS.

We report on a 6-year-old girl with 22q11DS presenting a form of epilepsy that can be classified as “Panayiotopoulos syndrome” (PS). To the best of our knowledge, PS has never been associated with 22q11DS. Array CGH revealed a *de novo* typical 22q11.2 microdeletion and a rare maternal microduplication of 172 kb in 2q37, which could represent an important factor predisposing to epilepsy. As in the case of CHD, additional CNVs outside the 22q11.2 region may modulate the risk of epileptic seizures in patients with 22q11DS.

## Case Report

The girl, the third child of healthy, unrelated parents, was born at full term after a normal pregnancy by cesarean section because of podalic presentation. Her mother had a previous miscarriage at 16 weeks of gestation. The neonatal course was regular. The patient came to our observation at 6 years of age because of recurrent episodes of vomiting. Her weight was 22.3 kg (−0.29 SD), height 118 cm (−0.61 SD), and BMI 16.6 (0.14 SD); she presented dysmorphic features such as a wide mouth with a “Cupid’s bow”-shaped upper lip, epicanthic folds, prognathism and narrow biparietal diameter, and a II–III toe syndactyly.

Parents reported several autonomic partial seizures in sleep (ictal vomiting, retching, pallor, and nausea) sometimes associated with unilateral eye deviation and sometimes with secondary generalization (loss of consciousness and urine incontinence). The episodes started at the age of 4; on that first occasion, she seemed completely unresponsive and flaccid for about 30 min. After that, she began to have episodes of paroxysmal vomiting during the day and then during the night, the frequency of which increased, starting from one episode every 3 months to one episode per month.

The child had also recurrent otitis; the audiological evaluation showed mild bilateral transmission hypoacusis.

She has a mild developmental delay and an attention deficit hyperactivity disorder. At 6 years, a psychometric scale WISC-III (I.Q. tot: 75, V.I.Q.: 70, and P.I.Q.: 85) was administered, which highlighted lower limits of intellectual quality, with a significant discrepancy between verbal and performance skills, with difficulty in accepting, detaining, and recalling verbal information.

During hospital admission, blood chemical and amino acid examinations were performed, as well as brain MRI, cardiac, and abdominal Doppler sonography; all of these test results were normal. In particular, the blood proline value (192 μmol/L) was in the normal range (n.v. 117–332 μmol/L).

Awake electroencephalographic (EEG) showed occipital spikes during eye closure with generalized spike wave complex during sleep. Considering the clinical features and the EEG pattern, we diagnosed an occipital early onset epilepsy syndrome, PS.

In accordance to the literature data, we decided not to use any antiepileptic drug.

The child underwent an immunologic consultation. A general lymphocyte overview showed normal values comparing to age-matched healthy controls; an extended evaluation of both T cell subpopulations by cytoflorimetric analysis showed that CD4+ and CD8+ effector memory (CD45RA−CD62L−) subset was abnormally high in the first examination.

An auxological and endocrinological consultation showed vitamin D deficiency and normal blood calcium levels (about 10 mg/dL, n.v. 8.6–10.2). The child did not assume supplements or other medications.

After written informed consent, a 180K oligo-array (Agilent, Santa Clara, CA, USA) was performed, according to standard procedures; the analysis was made with the Agilent dedicated software (Cytogenomics, Agilent). The Database of Genomic Variants (DGV) was used to define the variants found. Array CGH tests were also performed on her parents.

**Figure 1:**
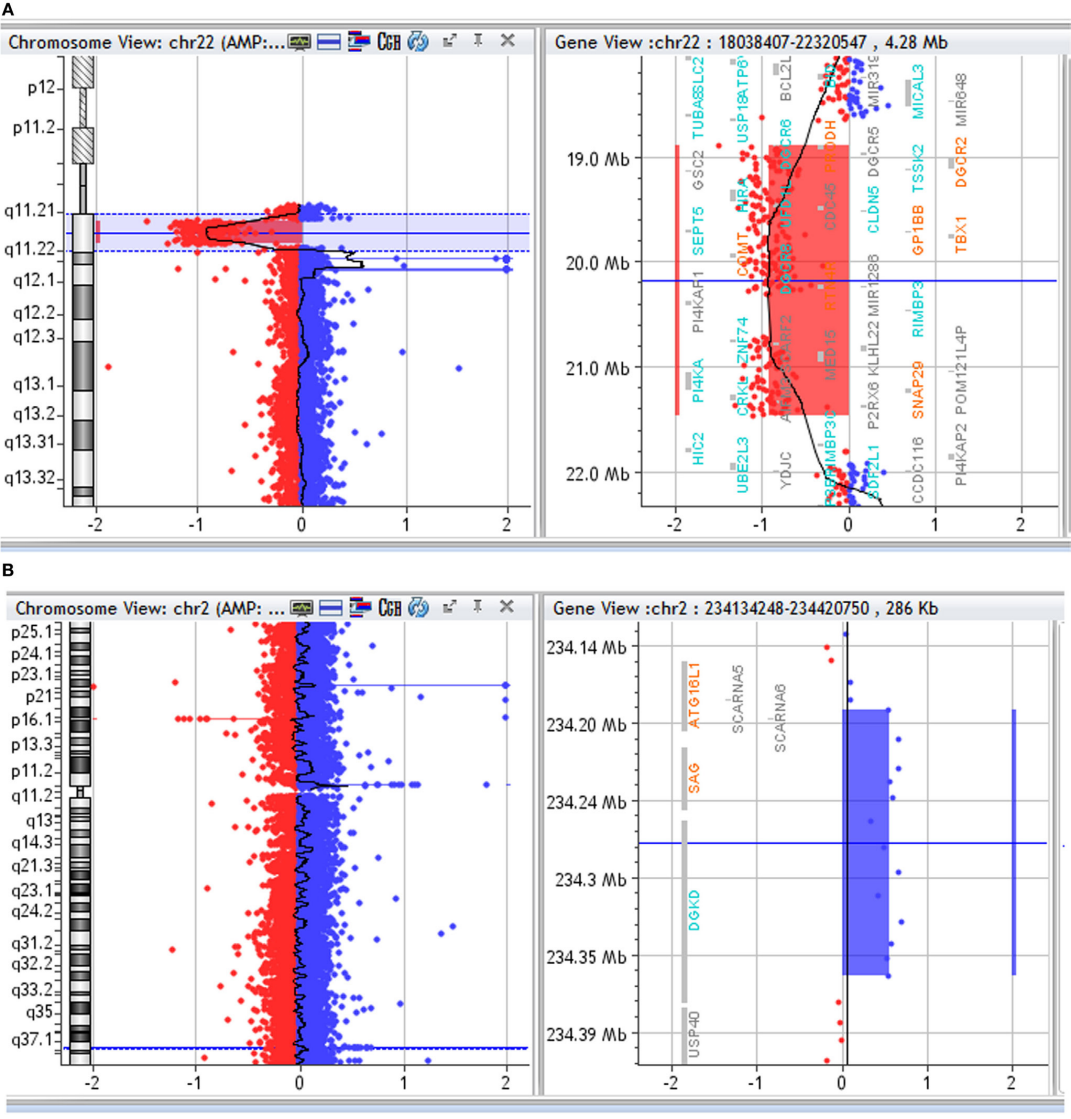
**(A)** Array CGH profile of chromosome 22. Array CGH of the proband showing chromosome 22 on the left and the 22q11.2 deletion highlighted on the right. **(B)** Array CGH profile of chromosome 2. Array CGH of the proband showing chromosome 2 on the left and the 2q37 duplication highlighted on the right.

Array CGH detected in the proband a *de novo* ~2.6 Mb deletion in 22q11.2, from position 18,894,835 to position 21,464,119 (hg19 map) and an additional 172 kb duplication in 2q37.1, from position 234,191,549 to position 234,363,450 (Figures 1A,B). FISH with a commercial probe (Cytocell, Tarrytown, NY, USA) was used to confirm the 22q11.2 deletion in the proband.

The 2q37.1 duplication was inherited from her healthy mother who did not present any neurological disease except for recurrent headaches.

**Figure 2:**
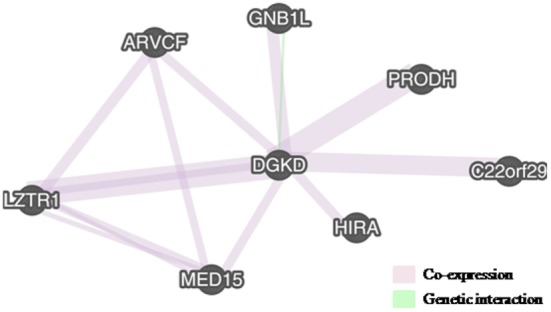
**Interactions between DGKD and the genes located in 22q11.2, as shown by GENEMania**. DGKD shows a co-expression with seven genes in 22q11.2 (PRODH, HIRA, GNB1L, C22orf29, ARVCF, MED15, and LZTR1) (pink lines) and functionally interacts with GNB1L (green line).

Putative interaction networks among genes harbored in the 22q11.2 and in the 2q37.1 region were built by the GeneMANIA^1^ with default parameters. Physical, co-expression, and pathway gene–gene interactions were evaluated (9). As shown in Figure 2, the network revealed that *DGKD* is co-expressed with *PRODH, HIRA, GNB1L, C22orf29, ARVCF, MED15*, and *LZTR1* and that it interacts with *GNB1L*. Details about the functional role of these genes are reported in Table 1.

**Table 1:** **Description of the functional role of the genes co-expressed with DGKD**.

Gene	Official name	REFSEQ ID	Function
PRODH	Proline dehydrogenase 1	*606810	This gene encodes a mitochondrial protein that catalyzes the first step in proline degradation. Mutations in this gene are associated with hyperprolinemia type 1 and susceptibility to schizophrenia 4 (SCZD4)
HIRA	Histone cell cycle regulation defective, *Saccharomyces cerevisiae*, homolog of, A	*600237	This gene encodes a histone chaperone that preferentially places the variant histone H3.3 in nucleosomes. This gene plays an important role in the formation of the senescence-associated heterochromatin foci. These foci likely mediate the irreversible cell cycle changes that occur in senescent cells. Insufficient production of the gene may disrupt normal embryonic development
C22orf29	Chromosome 22 open reading frame 29		Not characterized
ARVCF	Armadillo repeat gene deleted in velocardiofacial syndrome	*602269	ARVCF is a member of the catenin family. This family plays an important role in the formation of adherens junction complexes, which are thought to facilitate communication between the inside and outside environments of a cell
MED15	Mediator complex subunit 15	*607372	MED15 (PCQAP) is a component of the activator-recruited cofactor complex or the metazoan Mediator complex and is essential for TGFB/Activin/Nodal/Smad2/3 signal transduction
LZTR1	Leucine zipper-like transcriptional regulator 1	*600574	The LZTR1 gene encodes a protein that belongs to a functionally diverse superfamily of BTB/POZ (broad complex, tramtrack and bric-a-brac/poxvirus, and zinc finger) proteins. BTB-containing proteins control fundamental cellular processes, ranging from the regulation of chromatin conformation to the cell cycle
GNB1L	G protein subunit beta 1 like	*610778	This gene encodes a G-protein beta-subunit-like polypeptide, which is a member of the WD repeat protein family. Members of this family are involved in a variety of cellular processes, including cell cycle progression, signal transduction, apoptosis, and gene regulation

## Discussion

We report on a 6-year-old girl with 22q11DS presenting prolonged autonomic seizures (nausea, retching, ictal vomiting, pallor, and urine incontinence) that fits the criteria of PS.

Epilepsy is reported in 22q11DS, but few studies have focused on this topic, and most of them do not address epilepsy phenotype in more detail. In a recent article (5), a careful revision of the literature on 22q11DS patients with epilepsy and the description of six new cases have been made; among the 53 individuals reported in PubMed, 47% were diagnosed with focal epilepsy (including cases of Rolandic and atypical absence epilepsy), 41% had the diagnosis of genetic generalized epilepsy (including cases with myoclonic features and juvenile myoclonic epilepsy), 6% had myoclonic jerks and unspecified epilepsy, and 6% had unspecified epilepsy without myoclonic features. To the best of our knowledge, PS has never been associated with 22q11DS.

Epilepsy of the proband can be defined as “genetic” since neither structural CNS abnormalities nor metabolic conditions predisposing to epilepsy were found. In 22q11DS, “genetic epilepsy” has a prevalence of about 8.3%, suggesting that one or more genes in this specific locus may be associated to epileptogenesis (2).

This case shows the typical deletion between the low-copy repeats A and D in 22q11.2, where about 50 RefSeq genes are harbored. Neither ion channel genes nor genes whose hemizygous deletion causes epilepsy have been identified. The only gene that can be associated with epilepsy is *PRODH* (Proline Dehydrogenase 1), whose homozygous deletion causes type I hyperprolinemia, characterized by a high proline level in blood and various NP symptoms, including epileptic seizures (10). In this proband, *PRODH* has a functional allele since her proline level was normal.

However, it is worth noting that some other genes in the 22q11 deletion are involved in brain function and development, like *SNAP29* (synaptosomal-associated protein 29) and *RTN4R* (reticulon four receptor, NOGO RECEPTOR) (11, 12). A third gene, *DGCR8* (microprocessor complex subunit 8), encodes a subunit of the miRNA microprocessor complex and has a genome-wide effect on the biogenesis of micro-RNAs, which are particularly expressed in the mammalian brain (13). Therefore, it is possible that the hemizygous expression of multiple genes involved in the development of neuronal circuits can explain the broad spectrum of structural abnormalities and the coexistence of several NP manifestations, including epilepsy.

Since only a minority of patients experience epileptic seizures, the hemizygous deletion of 22q11 is not sufficient “*per se*” to cause epilepsy, and a “second hit” is necessary. In the present case, in addition to the 22q11 deletion, the array CGH data showed a 172-kb duplication in 2q37.1, where three genes are harbored: *ATG16L1* (Autophagy 16-like 1), *SAG* (S-antigen; retina and pineal gland), and *DGKD* (diacylglycerol kinase delta).

Several evidences support a role of *DGKD* in epileptogenesis.

*DGKD* encodes a cytoplasmic enzyme that phosphorylates diacylglycerol (DAG) to produce phosphatidic acid (PA). Both DAG and PA are important second messengers in a pathway of lipid signaling that has been implicated in epilepsy and other neurological diseases. Of special interest, in this respect, is DAG-gated transient receptor potential cation channels (e.g., TRPC3, -6, and -7), in which DAG binding directly opens the channels (14).

In *Drosophila* and mice, expression studies support a role for DGKD in development and function of CNS; EEG assessment of Dgkd knockdown mice revealed abnormal epileptic discharges in half of the mutant mice evaluated (15).

In humans, disruption of the *DGKD* sequence by a translocation between a chromosome X and a chromosome 2 has been previously reported in a patient with epilepsy (14).

Further data about human *DGKD* haploinsufficiency have been searched for in two publically available databases that collect pathogenetic imbalances in Decipher,^2^ ClinVar,^3^ and HGMD,^4^ a database that collects human genome mutations. This search did not give additional information, since the cases with only one imbalance involving *DGKD* (9 deletions and 4 duplications in Decipher, 7 deletions and 12 duplications in ClinVar) have a very large extent with an average size of several megabases, and their clinical description is very limited. No point mutations in *DGKD* are present in HGMD.

As for the 22q11.2 deletion, the 2q37.1 duplication, which leads to DGKD haploinsufficiency, is not “*per se*” sufficient to cause epilepsy. In the proband, this duplication is inherited from her mother, who never experienced epileptic seizures; a case with a duplication breaking *DGKD* sequence is reported in the DGV^5^ that collects CNVs identified in healthy controls (16).

We have searched for epistatic interactions between genes harbored in the two imbalances. *DGKD* shows a co-expression with six genes located in 22q11.2 (*PRODH, HIRA, C22orf29, ARVCF, MED15*, and *LZTR1*), and it functionally interacts with *GNB1L*, a gene involved in cell cycle progression, signal transduction, apoptosis, and gene regulation (Figure 2; Table 1).

Even if we cannot exclude that other smaller genetic variants or mutations elsewhere in the genome may play a role, it is likely that in this case the 2q37.1 duplication, which leads to *DGKD* haploinsufficiency, may represent the “second hit” necessary for the outcome of the epileptic phenotype.

As in the case of CHD, additional rare CNVs outside the 22q11.2 region may represent an important modifying factor underlying the variable expressivity and incomplete penetrance of epileptic seizures in 22q11DS.

## Concluding Remarks

Association between 22q11DS and epilepsy is evident; thus, a detailed description of the epileptic phenotype, when present, is mandatory. In larger cohorts of patients, an accurate analysis of the additional rare CNV gene content will be useful to elucidate the genetic mechanisms underlying the pathological phenotype.

## Ethics Statement

This study was approved by Pediatric Regional Ethic Committee (Comitato Etico Pediatrico Regionale per la sperimentazione clinica) of the AOU Meyer (Florence, Italy). Number of the folder: CGF-DEL 22q11.2 CR-AOUP. The patient signed an informed consent before genetic testing.

## Author Contributions

VB, AV, and AA performed array CGH analysis, contributed to the analyses and data interpretation, and wrote and critically revised the paper. AL and RC collected and interpreted the clinical data, performed the immunological studies, and contributed to the revision of the paper. GS contributed to the clinical collection of data and critically revised the paper. AO and AB performed the neurological assessment, interpreted the clinical data, and critically revised the paper.

## Conflict of Interest Statement

The authors declare that the research was conducted in the absence of any commercial or financial relationships that could be construed as a potential conflict of interest.

## Footnotes

^1^http://www.genemania.org/.

^2^https://decipher.sanger.ac.uk/.

^3^https://www.ncbi.nlm.nih.gov/clinvar/.

^4^http://www.hgmd.org.

^5^http://dgv.tcag.ca/.
